# Short pulse and directional thalamic deep brain stimulation have differential effects in parkinsonian and essential tremor

**DOI:** 10.1038/s41598-022-11291-9

**Published:** 2022-05-04

**Authors:** Ute Hidding, Miriam Schaper, Alessandro Gulberti, Carsten Buhmann, Christian Gerloff, Christian K. E. Moll, Wolfgang Hamel, Chi-un Choe, Monika Pötter-Nerger

**Affiliations:** 1 Department of Neurology, University Medical Center Hamburg-Eppendorf, Martinistraße 52, 20246 Hamburg, Germany; 2 Department of Neurosurgery, University Medical Center Hamburg-Eppendorf, 20246 Hamburg, Germany; 3 Department of Neurophysiology and Pathophysiology, University Medical Center Hamburg-Eppendorf, 20246 Hamburg, Germany

**Keywords:** Basal ganglia, Outcomes research, Neurology

## Abstract

The aim of this study was to assess the effects of novel stimulation algorithms of deep brain stimulation (short pulse and directional stimulation) in the ventrointermediate thalamus and posterior subthalamic area (VIM/PSA-DBS) on tremor in Parkinson’s disease (PD) and to compare the effects with those in essential tremor (ET). We recruited six PD patients (70.8 ± 10.4 years) and seven ET patients (64.4 ± 9.9 years) with implanted VIM/PSA-DBS in a stable treatment condition (> 3 months postoperatively). Tremor severity and ataxia were assessed in four different stimulation conditions in a randomized order: DBS switched off (STIM OFF), omnidirectional stimulation with 60 µs (oDBS60), omnidirectional stimulation with 30 µs (oDBS30), directional stimulation at the best segment with 60 µs (dDBS60). In both patient groups, all three DBS stimulation modes reduced the total tremor score compared to STIM OFF, whereas stimulation-induced ataxia was reduced by oDBS30 and partially by dDBS60 compared to oDBS60. Tremor reduction was more pronounced in PD than in ET due to a limited DBS effect on intention and action-specific drawing tremor in ET. In PD and ET tremor, short pulse or directional VIM/PSA-DBS is an effective and well tolerated therapeutic option.

**Trial registration: **The study was registered in the DRKS (ID DRKS00025329, 18.05.2021, German Clinical Trials Register, DRKS—Deutsches Register Klinischer Studien).

## Introduction

Parkinsonian (PD) and essential tremor (ET) are beyond the most common tremor syndromes^1–3^ with considerable impact on patients’ quality of life. In both disease conditions, deep brain stimulation (DBS) in the area of the ventral intermediate nucleus (VIM) and the posterior subthalamic area (PSA) is a good treatment option in case of drug therapy failure^4,5^. While the subthalamic nucleus (STN) or the globus pallidus internus (GPi) are often targeted in PD due to additional effects on bradykinesia and rigidity, VIM/PSA-DBS is preferred in elderly PD patients with lateralized tremor or slight cognitive impairment. VIM/PSA-DBS proved an effective treatment to suppress tremor, but its efficacy is limited by stimulation induced adverse effects^6–8^. In recent years, new forms of stimulation have expanded the therapeutic options. On the one hand, stimulation with short pulses (< 60 µs) is assumed to target specific nerve fibers along their different neurophysiological properties. On the other hand, a spatially restricted, more focal, directed electric field can be applied via segmented electrodes. For both stimulation modes, it has been recently demonstrated that the therapeutic window can be enhanced in ET patients with VIM/PSA-DBS^9–12^ or PD patients with STN-DBS^13,14^. To date, the use of novel stimulation algorithms in PD patients with VIM/PSA-DBS has not been assessed.

The pathophysiology of parkinsonian and essential tremor is quite different^15,16^. Parkinsonian tremor might be elicited by the combination of nigrostriatal degeneration and cerebello-thalamic circuit dysfunction. FDG-PET and functional MRI studies suggest that activity changes in the basal ganglia network involving the putamen and globus pallidus are associated to tremor onset while cerebellum, thalamus and the motor cortex correlate with tremor amplitude^16^. Thus it was proposed, that the basal ganglia circuit might trigger the cerebello-thalamo-cortical network to oscillate, resulting in tremor. In contrast, in ET the abnormality seems to be within the cerebello-thalamo-cortical network itself with a dysfunctional motor controller which sets off the oscillation^16^. The ventral intermediate nucleus (VIM) is a main relay nucleus embedded in the cerebello-thalamo-cortical circuit and an optimal target to interfere with circuit oscillations by DBS.

It is of interest, whether the effects of VIM/PSA-DBS depend on the preexisting pathophysiological state of the cerebello-thalamo-cortical circuit in these two disease conditions and whether the use of short pulse and directional stimulation can optimize potential effects and side effects in both tremor entities, parkinsonian and essential tremor. Thus, we aimed to evaluate the effect of short pulse and directional DBS in the VIM / PSA in PD patients and to compare DBS effects with those in ET patients.

## Materials and methods

### Study design

Six patients with tremor-dominant PD (all male) and seven ET-patients (six male) were included in this study. All but one PD patients were treated with unilateral thalamic DBS contralateral to the more affected side, all ET patients with bilateral thalamic DBS. Data of the ET patients have been published before^10^. One of the patients included in the former study was excluded from actual comparison because of newly diagnosed PD. For clinical details see Table 1. Written informed consent was obtained from all participants. The study protocol was approved by the Ethics Committee of the Hamburg Board of Physicians (PV5281). The assessments were conducted in accordance with the Declaration of Helsinki.

**Table 1:** Demographic data and stimulation parameters of Parkinsonian (PD) and Essential Tremor (ET) patients.

Patient	Sex	Age (years)	Disease duration (years)	Time after surgery (weeks)		Stimulation side	Stereotactic coordinates (mm) ventral contact			Stimulation amplitude (mA)	
							x	y	z	60 µs (ventral/dorsal contact)	30 µs (ventral contact)
**PD-patients**											
PD-1	M	77	5		15	Right	9.69	8.73	-5.4	3/3.5	4.2
PD-2	M	50	11		15	Right	11.87	7.96	− 1.87	2.5/4.5	3.5
PD-3	M	74	4		46	Right	11.91	6.76	− 2.81	2.8 3.3	3.9
PD-4	M	73	7		43	Left	13.28	8.54	− 1.56	3/-	4.2
PD-5	M	78	7		17	Left	10.99	9.69	− 5.17	3.8/4.3	5.4
						Right	9.76	9.38	− 5.43	3.1/3.5	4.4
PD-6	M	73	4		16	Left	10.82	6.65	− 2.97	4.5/-	6.4
**ET-patients**											
ET-1	F	77	17		25	Left	11.82	9.4	− 2.54	3.5/3	4.95
						Right	11.03	5.42	− 4.65	2.5/3	3.5
ET-2	M	74	60		18	Left	11.79	8.93	− 4.75	4/3.5	5.7
						Right	12.06	9.61	− 4.27	3.5/3.5	5
ET-3	M	58	12		15	Left	10.34	8.49	− 3.75	3/3.4	4.2
						Right	8.62	8.14	− 4.41	2.2/2.7	3.1
ET-4	M	72	55		34	Left	11.4	7.03	− 4.76	3/-	4.2
						Right	10.67	8.25	− 3.81	2.5/4	3.5
ET-5	M	53	20		15	Left	12.18	8.52	− 3.52	3.5/-	4.9
						Right	8.68	6.28	− 6.05	3/3.5	4.2
ET-6	M	54	35		21	Left	9.71	9.21	− 5.73	2.9/3.5	4.1
						Right	9.14	9.75	− 5.64	2.5/3	3.5
ET-7	M	63	36		26	Left	11.6	8.01	− 2.99	2.8/3	4
						Right	10.35	6.9	− 1.53	3.5/4.5	4.9

“Disease duration” (years) is calculated from symptom onset to the study assessment. “Time after surgery” (weeks) is calculated from the date of surgery to the study assessment. “Stimulation side” is the hemisphere side of the electrode. “Stereotactic coordinates” are the coordinates of the lowest “ventral” and 2nd lowest “dorsal” contacts with x = lateral to midline, y = posterior to mid-commissural point (MCP), z = above the plane between anterior and posterior commissure.

### Surgical procedure and definition of electrode position

All patients were implanted with the Infinity DBS system (Abbott Medical Neuromodulation Division, Plano, TX, USA) according to standard surgical procedures at our center as previously described in detail^17^. Based on preoperative T1 magnetic resonance imaging and post-operative computed tomography scans, the 2nd lowest contacts were located in the PSA. The optimal target site for electrode implantation was determined by multitrajectory microelectrode recordings (BenGun, up to three microelectrodes) and clinical evaluation of macrostimulation effects as described previously^18^. Coordinates of the electrode contacts were determined from the artifact in postoperative CT scans (Brainlab, Munich, Germany) in relation to the mid-commissural plane (x = medio-lateral, y = anterior–posterior, z = ventro-dorsal to mid-commissural point).

### Stimulation parameters

**Figure 1:**
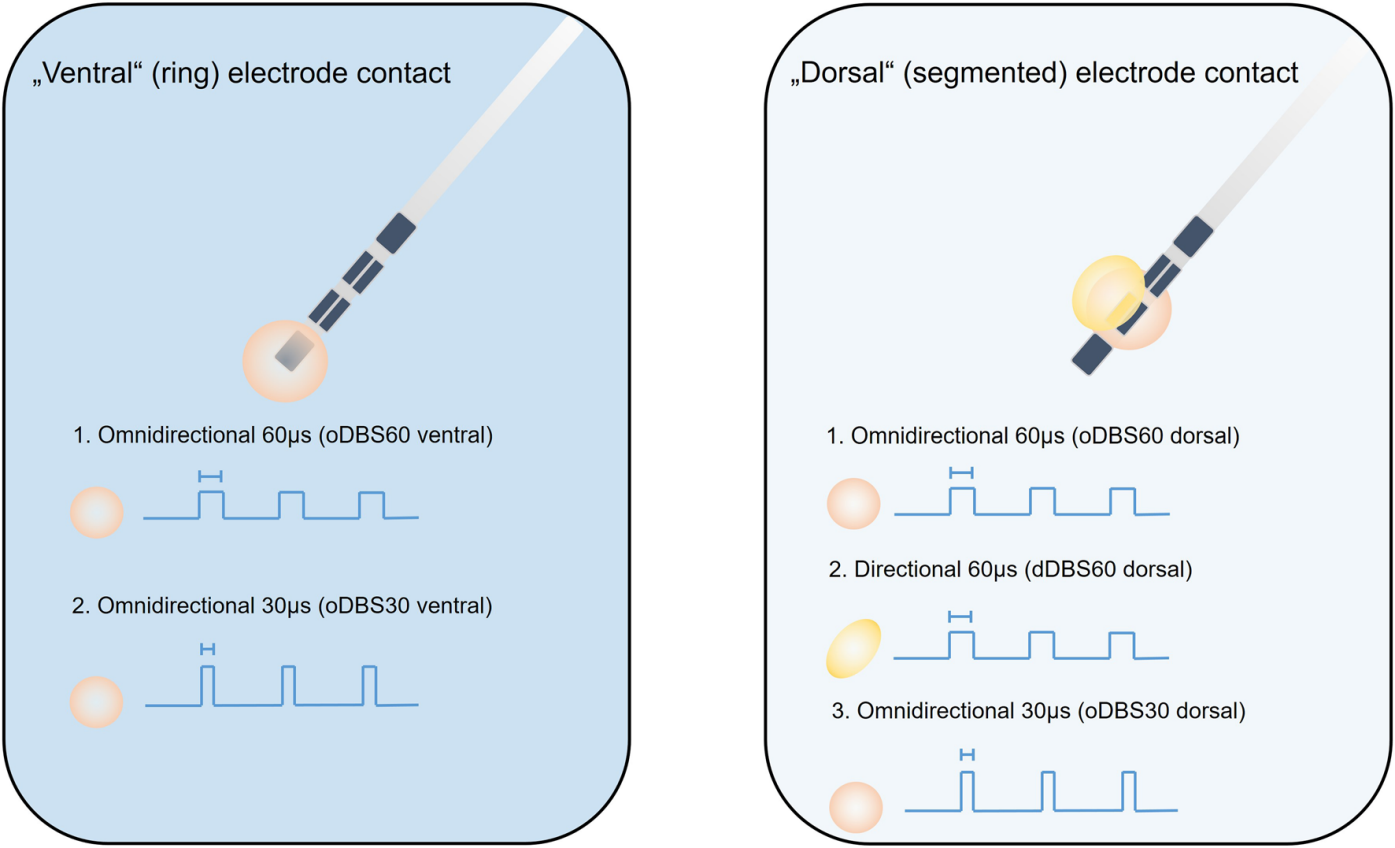
Study design. Parkinsonian (PD) and essential Tremor (ET) patients were studied in the following experimental conditions: (1) stimulation at the lowest, most ventral contact in an omnidirectional stimulation mode with a pulse width of 60 µs and 30 µs (left column), (2) Stimulation at the second lowest, dorsal, segmented contact with omnidirectional and directional stimulation at one single segment with a pulse width of 60 µs (right column).

As ataxia occurs mainly by ventral stimulation at distal contacts, patients were assessed in the following stimulation conditions (Fig. 1): (1) no stimulation (OFF), (2) on the lowest, ventral contact two stimulation modes were applied: (2.1) Omnidirectional, suprathreshold stimulation with 60 µs pulse width and clinical signs of dysmetria and ataxia (*oDBS60 ventral*), (2.2) Omnidirectional stimulation with the same energy dose equivalent amplitude at constant frequency but applied with 30 µs (*oDBS30 ventral*). On the 2nd lowest, dorsal contact three stimulation modes were used: (3.1) Omnidirectional suprathreshold stimulation with 60 µs and clinical signs of dysmetria and ataxia (*oDBS60 dorsal*), (3.2) Directional stimulation with 60 µs and the same amplitude as omnidirectional stimulation (d*DBS60 dorsal*), (3.3) Omnidirectional stimulation with the same energy dose equivalent amplitude at constant frequency but applied with 30 µs (*oDBS30 dorsal*) (Fig. 1). Thus, short pulse stimulation was assessed on the two lowest contacts and directional stimulation on the second lowest contact only. The required stimulation amplitude was determined by incremental amplitude increases of 0.5 mA with a pulse width of 60 µs until the onset of ataxia and subsequent reduction in 0.1 mA steps as described earlier^19^. Suprathreshold stimulation was defined as stimulation amplitude 0.5 mA above the threshold for inducing ataxia. The total energy delivered (TEED) was calculated according to:

$$TEED=\frac{current2\times frequency\times pulse \; width}{impedance}\times 1 \; second$$

assuming a standard impedance of 1000 Ω^20^. Only the best segment was included in the analysis, which was defined as the segment with the best effect on tremor at the suprathreshold amplitude or—in case of equal tremor effect—with the least induction of ataxia. Electrode contacts without ataxia side effects were excluded from the analysis (2 of 14 in PD, 4 of 28 in ET). Thus, the analyzed number for short pulse stimulation was n = 7 (PD) and n = 14 (ET), and for directional stimulation n = 5 (PD) and n = 10 (ET), respectively.

### Clinical assessment

Severity of limb tremor was evaluated using the Fahn-Tolosa-Marin Tremor Rating Scale (FTMRS) using items 5 or 6 (rest, postural and action-/intention tremor of right or left upper extremity), 11 (drawing large Archimedes spiral), 12 (drawing small Archimedes spiral) and 13 (drawing straight lines). FTMRS hand subscore was calculated by summing up items 5/6, 11, 12 and 13 (maximum score 24 with higher scores indicating more tremor). Severity of limb ataxia was evaluated using item 10 and 14 of the International Cooperative Ataxia Rating Scale (ICARS). ICARS hand subscore was calculated by summing up items 10 and 14 (maximum score 8 with higher scores indicating more severe dysmetria and ataxia). Paresthesia was evaluated using a visual analog scale (0—none, 10—strongest sensation). For comparison with preoperatively collected UPDRS data, a sum score from the tremor items for the upper extremity of the MDS-UPDRS III was also calculated in PD patients (item 3.15–3.18). In ET patients, the FTMRS was collected preoperatively. Patients and examiners were blinded for the stimulation conditions which were randomized by a second investigator. The clinical assessment was done 5–10 min after changing of stimulation parameters.

### Statistical analysis

All data are given as mean ± standard deviation (SD). Shapiro-Wilks tests were calculated to confirm normal distribution, in case of not-normally distributed data, data were transformed logarithmically. In a first step, PD patients were analyzed as a separate group. GLM ANOVA with the intrasubject factor stimulation condition (pulse width or directional/omnidirectional stimulation) was performed for the main symptom domains tremor, ataxia and paresthesia. In a second step, GLM ANOVA with the intrasubject factor stimulation condition (pulse width or directional/omnidirectional stimulation) and the intersubject factor disease condition (PD vs. ET) was performed. Greenhouse Geisser correction was used, if sphericity was violated. Post-hoc 2-tailed paired t-tests were used to specify intrasubject effects. As this was an exploratory study, group effects (PD vs. ET) were analysed with uncorrected, unpaired t-tests. Statistical analyses were performed with SPSS (IBM SPSS Statistics for Windows, version 20, SPSS Inc., Chicago, IL, USA) and GraphPad Prism (version 9 for Windows, La Jolla, USA).

## Results

### Subject characteristics

Detailed group characteristics are summarized in Table 2. ET and PD-groups did not differ in terms of age or time since surgery. Mean age was 70.8 ± 10.4 years (PD patients) and 64.4 ± 9.9 years (ET patients), mean duration of DBS treatment was 174.8 ± 102.7 days (PD patients) and 162.6 ± 52.6 days (ET patients). ET patients showed a significant longer disease duration (PD: 6.8 ± 3.1 years; ET: 33.6 ± 18.7 years; p = 0.002). The position of the electrodes did not differ significantly between PD and ET patients. Given the border between VIM and PSA at the level of the midcommissural line, all but one contact investigated were located in the PSA. The stimulation amplitude to induce ataxia tended to be higher in PD patients, but the difference was not significant (PD 3.58 ± 0.68 mA. ET 3.19 ± 0.53 mA, p = 0.06).

**Table 2:** Group characteristics of PD and ET patients.

	PD	ET	p
Age (years)	70.8 (± 10.42)	64.43 (± 9.91)	0.28
Disease duration (years)	6.33 (± 2.66)	35.57 (± 18.66)	**0.0047**
Time after surgery (days)	174.8 (± 102.68)	162.57 (± 52.55)	0.79
Interval pre-post-DBS examination (days)	455.83 (± 204.32)	344.71 (± 129.27)	0.26
**Stereotactic coordinates (mm) Lowest “ventral” contact**			
x	11.25 (± 1.27)	10.65 (± 1.25)	0.35
y	8.51 (± 0.96)	8.14 (± 1.30)	0.55
z	− 3.71 (± 1.67)	− 4.17 (± 1.27)	0.52
**Stereotactic coordinates (mm) 2nd lowest “dorsal” contact**			
x	11.92 (± 1.07)	11.56 (± 1.17)	0.57
y	7.01 (± 0.88)	6.65 (± 1.26)	0.58
z	− 1.91 (± 1.33)	− 2.09 (± 1.27)	0.80
Stimulation amplitude (mA) for induction of ataxia with 60 µs	3.58 (± 0.68)	3.19 (± 0.53)	**0.06**

Significant values are in bold.

“Disease duration” (years) is calculated from symptom onset to the study assessment. “Time after surgery” (days) is calculated from the date of surgery to the study assessment. “Interval pre-post-DBS examination” (days) is calculated from the date of the pre-operative evaluation to the study assessment. “Stereotactic coordinates” (mm) are the coordinates of the “ventral” lowest and “dorsal” 2nd lowest contacts with x = lateral to midline, y = posterior to mid-commissural point (MCP), z = above the plane between anterior and posterior commissure. “Stimulation amplitude” (mA) is the suprathreshold amplitude to induce ataxia with 60 µs pulse width in ring mode.

### Perioperative tremor scores in PD and ET patients

**Figure 2:**
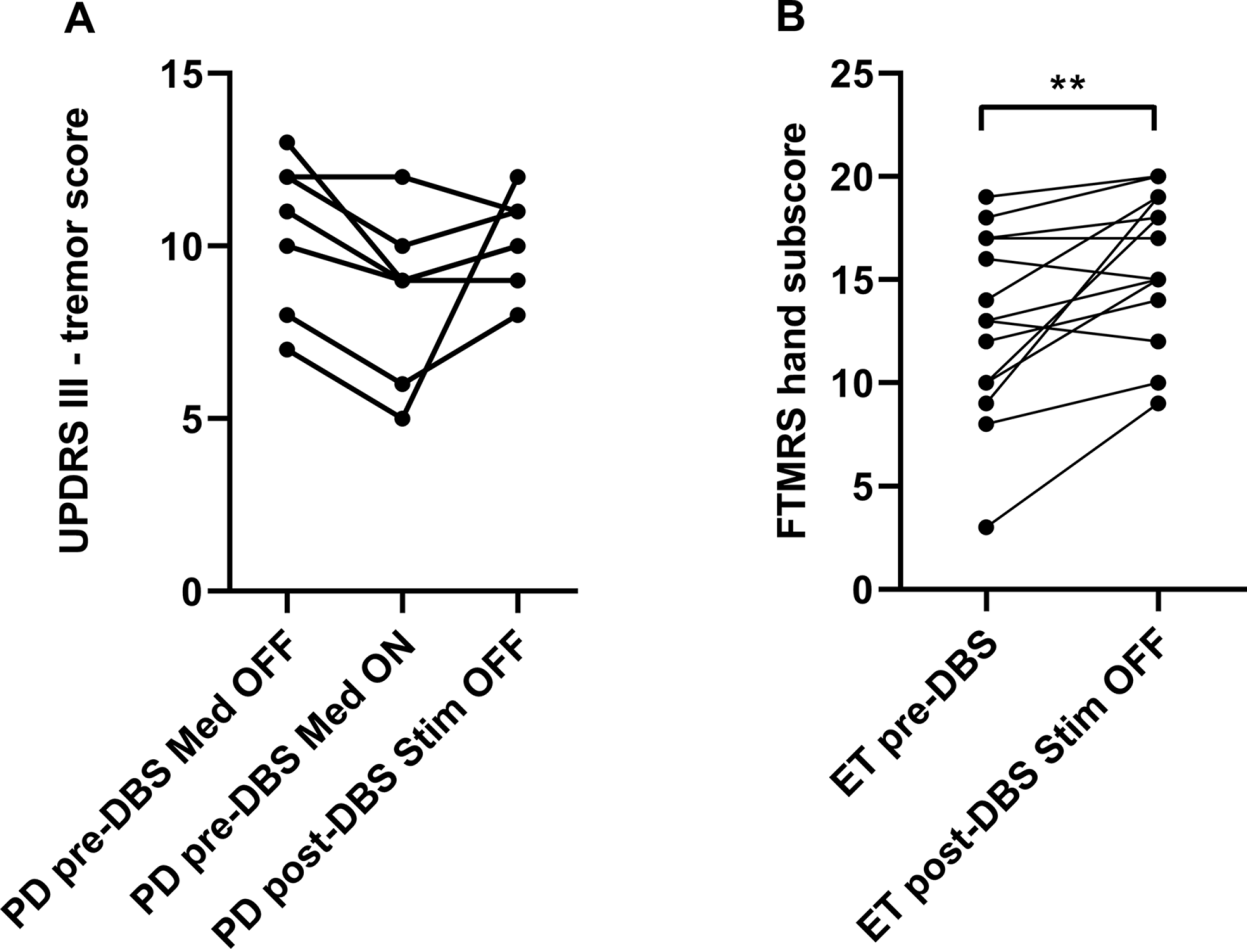
Tremor scores pre- and postoperatively without DBS. (**A**) Parkinsonian (PD) patients were assessed by the tremor items of the UPDRS preoperatively in Med OFF and Med ON condition as well as postoperatively in Med ON STIM OFF. (**B**) Essential tremor (ET) patients were assessed pre- and postoperatively in Med OFF condition by the FTMRS. In STIM OFF, PD tremor remained stable, whereas the tremor intensity in ET worsened during the postoperative course indicating natural disease progression. **Significance level of p < 0.01. Figure was created with GraphPad Prism (version 9 for Windows, La Jolla, USA).

The course of pre- and postoperative tremor scores in the observation period of about 6 months differed between both patient groups (Fig. 2). In PD patients, there was a slight, but not significant dopaminergic tremor response in a preoperative L-Dopa challenge using suprathreshold morning dosage (UPDRS-tremor score: preoperatively Med OFF 10.4 ± 2.2, Med ON 8.6 ± 2.4). The pre- and postoperative STIM OFF tremor scores at the time of the experiment were comparable (UPDRS III tremor score: pre-DBS Med OFF 10.4 ± 2.2, post-DBS STIM OFF 10.14 ± 1.35, p = 0.10) (Fig. 2A) indicating stable tremor characteristics in the off condition in the observation period. In contrast, tremor scores of ET patients significantly worsened in the longitudinal time course with significant perioperative increase of tremor severity (FTMRS sum score: pre-DBS 12.79 ± 4.49, post-DBS 15.79 ± 3.58 (p = 0.005; Fig. 2B). Noteworthy, the interval between pre- and postoperative assessments did not differ in PD and ET patients (Table 2).

### Conventional and new stimulation modes of VIM/PSA-DBS in PD patients

In PD patients, new stimulation forms as short pulse stimulation and directional stimulation were compared to conventional omnidirectional 60 µs VIM/PSA-DBS.

The use of *short pulse stimulation* was equivalent in suppressing tremor compared to conventional VIM/PSA-DBS, but with less side effects at the ventral and adjacent dorsal contact. GLM ANOVA with the factor stimulation mode (3 levels: 1. STIM OFF 2. oDBS60 3. oDBS30) revealed significant effects for the tremor hand subscore (ventral contact: F = 36.66, p = 0.001; dorsal contact: F = 29.29, p = 0.004) and ataxia (ventral contact: F = 36.86, p < 0.001, dorsal contact: F = 15.65, p = 0.002). Paresthesia was not significantly different during the different STIM conditions.

**Figure 3:**
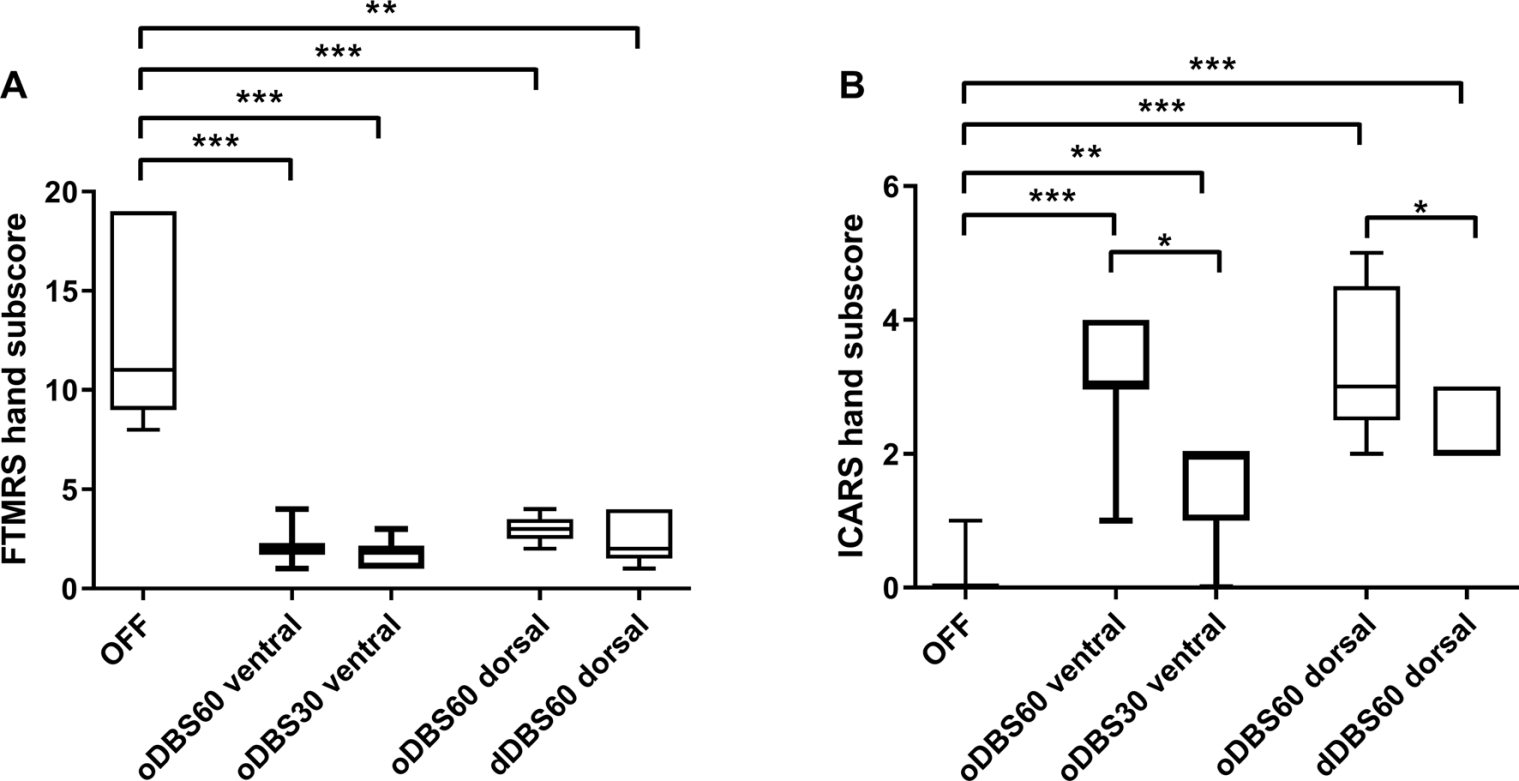
Effects of new DBS stimulation modes in PD patients. (**A**) Effect of the different DBS stimulation modes on tremor intensity measured by FTMRS. (**B**) Effect of the different DBS stimulation modes on ataxia assessed by ICARS. Stimulation modes: *oDBS60 ventral:* omnidirectional stimulation with 60 µs pulse width at the most ventral electrode contact, *oDBS30 ventral:* omnidirectional stimulation with the same energy dose equivalent amplitude at constant frequency with 30 µs at the most ventral electrode contact, *oDBS60 dorsal*: omnidirectional stimulation with 60 µs at the adjacent, second lowest, dorsal contact, *dDBS60 dorsal:* directional stimulation with 60 µs and the same amplitude as for omnidirectional stimulation at the dorsal contact. Asterisks indicate the different significance levels *p < 0.05, **p < 0.01, ***p < 0.001). All stimulation modes reduced tremor intensity compared to STIM OFF. The stimulation mode oDBS30 ventral induced less ataxia compared to oDBS60 ventral. Figure was created with GraphPad Prism (version 9 for Windows, La Jolla, USA).

The divergent clinical effects and side effects were most evident at the ventral contact, but also observed at the dorsal contact. The tremor hand subscore was significantly improved by both oDBS60 (ventral 2.14 ± 0.9; p = 0.001, dorsal 3.0 ± 0.7; p = 0.004) and oDBS30 (ventral 1.71 ± 0.76 p = 0.001, dorsal 2.0 ± 1.22 p = 0.006) compared to STIM OFF (12.86 ± 4.6), there was no significant difference between both stimulation modes indicating comparable tremor improvement by both stimulation modes in PD (Fig. 3A). However, both stimulation modes differed in terms of side effects. The use of conventional oDBS60 induced significant ataxia (ventral 3.0 ± 1.0 p < 0.001, dorsal 3.4 ± 1.14 p = 0.003), which was not present in STIM OFF (0.14 ± 0.38). Stimulation-induced ataxia was significantly less accentuated while oDBS30 (ventral 1.43 ± 0.78 p < 0.001, dorsal 1.2 ± 1.3 p = 0.04) compared to oDBS60 indicating a better side effect profile using short pulses (Fig. 3B).

The use of *directional stimulation* at the adjacent, 2nd lowest, “dorsal” contact revealed a similar pattern when comparing omnidirectional and directional stimulation using the same pulse width. The tremor hand subscore was significantly reduced (GLM ANOVA factor STIM condition F = 29.41, p < 0.001) by both stimulation forms (oDBS60 dorsal 3.0 ± 0.7, p = 0.001; dDBS60 dorsal 2.60 ± 1.34, p = 0.006) compared to STIM OFF (12.86 ± 4.6) with no significant differences between both stimulation modes (Fig. 3A). In terms of the side effect ataxia (GLM ANOVA STIM F = 36.91, p = 0.004), directional stimulation at the best segment (dDBS60 2.40 ± 0.55) induced significantly less ataxia than omnidirectional DBS (oDBS60 dorsal: 3.40 ± 1.14, p = 0.006) (Fig. 3B).

In a last step, we compared the efficiency of oDBS60, oDBS30 and dDBS60 at the dorsal contact in PD patients by analyzing the relative differences of symptom scores compared to STIM OFF. The improvement of the tremor hand subscores was similar between different stimulation modes (GLM ANOVA factor STIM condition F = 1.0, p = 0.405) with no significant differences between the degree of tremor improvement in post-hoc t-tests. In contrast, in terms of the side effect ataxia there were differences (GLM ANOVA STIM F = 4.61, p = 0.047). Stimulation-induced ataxia was significantly less accentuated during oDBS30 (1.2 ± 1.3) compared to oDBS60 (3.4 ± 1.14; p = 0.04); besides oDBS30 was slightly, but not significantly better compared to dDBS60 (2.4 ± 0.55, p = 0.18).

### Comparison of VIM/PSA-DBS effects between PD and ET patients

#### Comparison of tremor effects with new stimulation modes

The FMTRS hand subscore was significantly reduced by VIM/PSA-DBS at the ventral and dorsal contact in both patient groups regardless of the *pulse width*. GLM ANOVA with the intrasubject factor stimulation condition (3 levels: 1. STIM OFF 2. STIM ON 60 µs 3. STIM ON 30 µs) and the intersubject factor disease condition (2 levels: 1. PD 2. ET) revealed a highly significant difference for the factor stimulation condition (ventral contact: F = 113.31, p < 0.001; dorsal contact: F = 62.58, p < 0.001), but no significant interaction between stimulation condition and disease. Comparable to PD patients, the tremor hand subscore was significantly reduced in ET patients in both STIM ON conditions (OFF: 15.79 ± 3.58, oDBS60 ventral: 6.00 ± 3.16, oDBS60 dorsal: 6.2 ± 3.77 vs. oDBS30 ventral 5.0 ± 3.01, oDBS30 dorsal 4.9 ± 3.54 p < 0.0001) with no significant differences between STIM ON conditions (Supp. Table 1).

*Directional* and omnidirectional DBS were also equally effective in the suppression of the tremor hand subscore in both patient groups at the adjacent dorsal, segmented contact. GLM ANOVA revealed a significant effect for stimulation condition (F = 60.442, p < 0.001), but no significant interaction with disease condition indicating similar trends in ET and PD.

**Figure 4:**
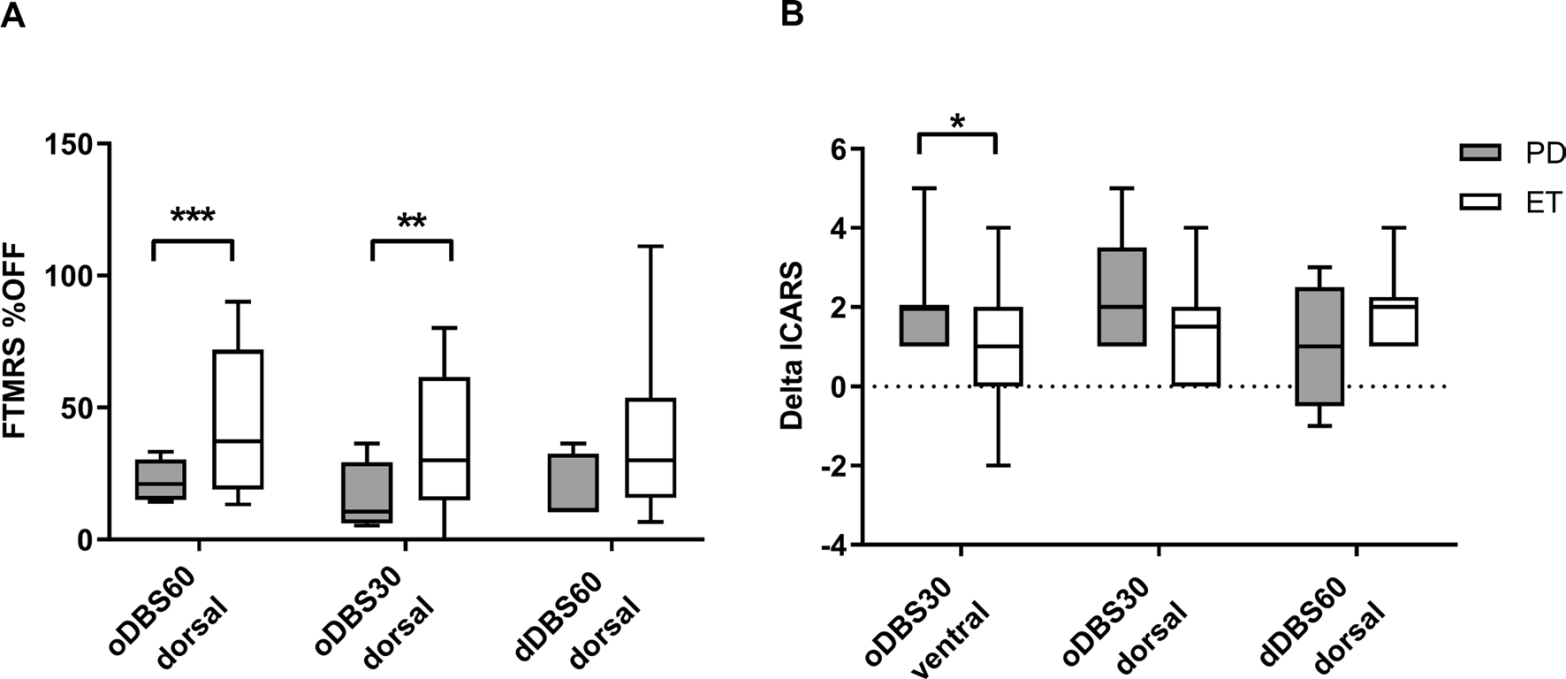
Comparison of new DBS stimulation modes between PD and ET patients. (**A**) Tremor score was normalized to the OFF FTMRS-Score. Both oDBS60 and oDBS30 at the dorsal contact reduced tremor in PD more than in ET patients. (**B**) Difference between the ataxia sum score at oDBS60 and the new DBS stimulation modes oDBS30 and dDBS60 at the corresponding electrode. In PD patients, oDBS30 reduced ataxia more than in ET patients at the ventral stimulation level. There was no significant difference for oDBS30 dorsal and dDBS60. Asterisks indicate the different significance levels *p < 0.05, **p < 0.01, ***p < 0.001). Figure was created with GraphPad Prism (version 9 for Windows, La Jolla, USA).

However, focusing on disease specific effects in more detail by normalizing the total tremor scores in STIM ON in relation to the STIM OFF condition with lower percentages indicating better tremor reduction, we observed differential effects of DBS in both patient groups. The relative tremor reduction was more pronounced in PD patients for all stimulation modes compared to ET. This effect was significant for oDBS60 (oDBS60 ventral: PD 19.66% ± 7.2, ET 40.46% ± 22.5, p = 0.0003; oDBS60 dorsal: PD 22.35% ± 7.98, ET 43.28% ± 27.7, p = 0.048) and oDBS30 at the ventral site (oDBS30 ventral: PD 15.86% ± 10.63, ET 31.18% ± 22.64, p = 0.0024, oDBS30 dorsal: PD 16.3% ± 13.0, ET 35.13% ± 26.36, p = 0.16), and approached significance for directional stimulation (dDBS60 dorsal: PD 19.42% ± 12.23, ET 42.24% ± 33.96, p = 0.08) (Fig. 4A).

To examine this effect in more detail, we had a closer look at the different tremor entities as tremor at rest, postural tremor, action and intention tremor and tremor while drawing Archimedes spiral.

**Figure 5:**
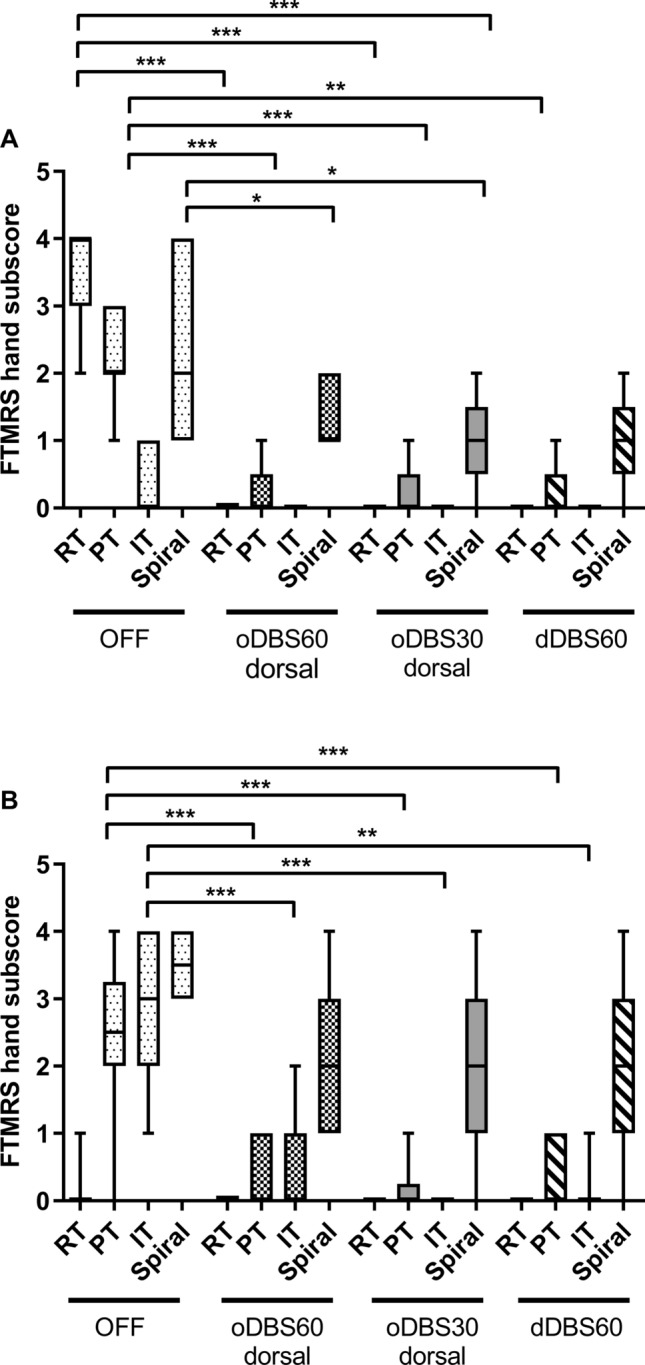
Response of tremor subtypes to the different stimulation conditions in PD and ET. *RT:* rest tremor, *PT*: postural tremor, *IT*: action / intention tremor, *Spiral*: small spiral (item 12 of the FTMRS). Note that data are only shown for the dorsal level for all conditions. Asterisks indicate significance level ***p < 0.001*.* Figure was created with GraphPad Prism (version 9 for Windows, La Jolla, USA).

For *short pulse stimulation,* a two factorial GLM ANOVA with the intrasubject factor STIM condition and tremor subtype revealed for both contacts a highly significant effect for STIM (ventral: F = 179.13, p < 0.001, dorsal: F = 7.16, p = 0.011) and tremor subtype (ventral: F = 52.01, p < 0.001; dorsal: F = 33.19, p < 0.001) with significant interaction of tremor subtype with disease condition (ventral: F = 28.56, p < 0.001; dorsal: F = 5.71, p = 0.013) and tremor subtype with STIM condition (ventral: F = 26.15, p < 0.001; dorsal: F = 11.0, p < 0.001) indicating a tremor subtype specific action of short pulse and conventional DBS. In detail, the predominant tremor at rest and postural tremor in PD patients was completely suppressed by VIM/PSA-DBS, whereas intentional and action tremor, particularly drawing spirals, was less impacted by VIM/PSA-DBS, which was more prominent in ET patients (Fig. 5).

In terms of *directional stimulation,* there was again a highly significant effect for the factor stimulation (F = 32.89, p < 0.001), tremor subtype (F = 21.26, p < 0.001) with significant interaction with disease condition (F = 9.7, p = 0.002) as well as with stimulation mode and disease condition (F = 4.51, p = 0.019) revealing tremor-specific action of the different stimulation modes in both disease entities. Again, the predominant resting tremor in PD patients was improved by omni- and directional stimulation. In ET patients, the postural and intention tremor was improved, but the predominant drawing tremor was less impacted by both stimulation modes (Fig. 5).

In a last step, we compared in both patient groups the efficiency of conventional, short pulse and directional stimulation at the best segment at the dorsal contact, using the relative differences of total tremor hand subscores compared to STIM OFF. The improvement of the total tremor hand subscores was distinct between oDBS60, oDBS30 and dDBS60 in the total cohort of both PD and ET patients (GLM ANOVA factor STIM condition F = 3.7, p = 0.039) with the significantly best effect with oDBS30 compared to oDBS60 (p = 0.006) and dDBS60 (p = 0.041).

#### Comparison of side effects with new stimulation modes

Suprathreshold stimulation with 60 µs triggered ataxia as measured by the ICARS sum score (PD: OFF: 0.14 ± 0.38, ventral: oDBS60 3.0 ± 1.0, dorsal: oDBS60 3.40 ± 1.14; ET: OFF: 0.14 ± 0.36, ventral: oDBS60 2.86 ± 1.35, dorsal: 3.40 ± 1.35). GLM ANOVA with the intrasubject factor stimulation condition (3 levels: 1. STIM OFF 2. STIM ON 60 µs 3. STIM ON 30 µs) and the intersubject factor disease condition (2 levels: 1. PD 2. ET) revealed a highly significant effect for the factor stimulation condition (ventral: F = 77.55, p < 0.001, dorsal: F = 33.58, p < 0.001), but no significant interaction between stimulation condition and disease. Post-hoc analyses revealed that ataxia induced by conventional 60 µs could be significantly reduced by usage of short pulse stimulation with 30 µs in both patient groups (PD: ventral: 1.43 ± 0.78; p = 0.001, dorsal: 1.2 ± 1.30, ET: ventral: 2.29 ± 0.64; p = 0.001, dorsal: 2.0 ± 2.0).

Likewise, best segment stimulation at the adjacent, dorsal contact induced less ataxia than omnidirectional stimulation. GLM ANOVA with the intrasubject factor stimulation condition (3 levels: (1) STIM OFF (2) STIM ON 60 µs (3) STIM ON best segment 60 µs) at the adjacent dorsal contact and the intersubject factor disease condition revealed again a highly significant effect for the factor stimulation condition (F = 82.48, p < 0.001), but no significant interaction between stimulation condition and disease.

We compared disease conditions more in detail by calculating the absolute difference between the ICARS sum score with oDBS60 ventral and the new stimulation condition (DeltaICARS), with larger values indicating less ataxia with the new stimulation condition. The use of oDBS30 resulted in better ataxia reduction in PD patients compared to ET patients at the ventral site (DeltaICARS oDBS30 ventral: PD: 1.83 ± 1.11, ET 0.92 ± 1.32 p = 0.040; dorsal: PD: 2.2 ± 1.64, ET 1.4 ± 1.26 p = 0.31), while the use of dDBS60 dorsal showed no significant difference between patient groups (DeltaICARS: dDBS60 dorsal: PD 0.80 ± 1.5, ET 1.86 ± 0.86, p = 0.17) (Fig. 4B).

The comparison of the efficiency of conventional, short pulse and directional stimulation at the best segment at the dorsal contact in both patient groups, using the absolute differences of ataxia revealed in both groups significant differences by the stimulation modes (GLM ANOVA STIM F = 12.52, p < 0.001). In the whole cohort with both ET and PD patients, oDBS30 induced significantly less ataxia than oDBS60 (p < 0.001) and even than dDBS60 (p = 0.009). The use of dDBS60 was associated with slightly less ataxia than oDBS60, but did not reach significant levels (p = 0.072).

## Discussion

The aim of this study was to assess the effects of new stimulation algorithms of VIM/PSA-DBS on tremor in PD patients and compare the effects with those in ET patients. The different tremor characteristics of the two patient groups have to be considered in the interpretation of VIM/PSA-DBS effects. Within the observation period of about 6 months, there was no natural progression of tremor severity in PD patients, but in ET patients in the STIM OFF condition. However, potential rebound effects of tremor reoccurrence after switching off stimulation should be considered, which could partly explain the tremor deterioration in ET patients observed here. In both patient groups, all three DBS stimulation modes reduced equally the total tremor score compared to STIM OFF, whereas the side effect profile in terms of stimulation-induced ataxia was better during oDBS30 and by dDBS60 compared to oDBS60. Stimulation-induced tremor reduction was more pronounced in PD than in ET due to a limited DBS effect on intention and action-specific drawing tremor, which was more prominent in ET.

There are quite well-known effects of conventional VIM/PSA-DBS in parkinsonian and essential tremor. The conventional settings of VIM/PSA-DBS are usually a continuous delivery of pulses with a frequency of 130 Hz and 60 µs pulse width. In a large European, multicenter study^4^, upper and lower limb tremor scores were improved in both patient groups. Tremor at rest was reduced by 85% in parkinsonian patients and postural tremor was decreased in 89% the ET patients associated with improvement of activities of daily living in both groups^4^. Parkinsonian tremor has been treated in the last decades by DBS in various targets as globus pallidus, subthalamic nucleus, pedunculopontine nucleus or caudal zona incerta. Nevertheless, in a recent metaanalysis the VIM has been proven to be the better target for Parkinsonian tremor reduction in the medication off condition^21^. A review of VIM-DBS effects in ET patients reported an improvement of tremor scores particularly of upper limb tremor by 90%^22^. Although conventional VIM-DBS is quite beneficial in the treatment of tremor, there are limitations of that therapeutical approach, particularly in terms of side effects as ataxia or paresthesia, which narrow the therapeutic window and require DBS reprogramming and trouble-shooting.

Particularly the induction of ataxia by stimulation of the ventral VIM or the adjacent PSA has recently become a focus of interest. It has been demonstrated that DBS of PSA is more effective in improving tremor than the VIM itself^5,8^. The anatomical background is that cerebello-thalamo-cortical fibers of the PSA are bundled together in a „bottleneck”, so that even low stimulation amplitudes excite all target fibers^8^. In contrast, ventral PSA stimulation induced more often unintentional side effects as ataxia, which was less pronounced while stimulating the more dorsal contacts^23,24^. This syndrome can occur as an acute effect but particularly gait ataxia can also manifest several months after an initially effective therapy and necessitate frequent adjustments of stimulation parameters^24^. In MR based studies, the fibers involved in tremor suppression and induction of ataxia could be further characterized. The fibers arriving from the dentate nucleus of the contralateral cerebellum, travelling through the bottleneck to the thalamus and the motor cortex seemed to be the target fibers for tremor reduction. In contrast, cerebello-rubro-spinal fibers might be possibly involved in the induction of ataxia by suprathreshold stimulation^23,24^. This close vicinity of fibers mediating beneficial effects for tremor suppression and fibers involved in pathogenesis of unintentional side effects, necessitates the use of specific DBS settings precisely targeting dentato-cerebello-thalamic fibers and preventing stimulation of cerebello-rubro-spinal fibers.

Recently, the introduction of new DBS devices enabled the release of short pulse stimulation and the possibility to steer the current by directional stimulation through segmented leads. As the DBS systems allow the modification of pulse width in 10 µs steps, in clinical studies 30 µs as used in this study or 40 µs have been applied^10,11,14,25,26^. So far, there is no evidence of a clear clinical advantage of 30 µs compared to 40 µs.

Short pulse DBS within the STN revealed in PD patients an increased therapeutic window and a reduction of unintentional side effects such as dysarthria^13,14,27,28^. Besides, this new stimulation option was assumed to be energy-saving compared to DBS with longer pulses^11,13,14,27,28^. Furthermore, short pulse VIM/PSA-DBS in essential tremor patients was shown to be clinically advantageous with lower incidence of side effects as paresthesia, hand- and gait ataxia^10,25^. The effect of short pulse and directional stimulation in the VIM/PSA on tremor and ataxia in PD patients was not assessed so far.

The potential mechanisms of action of short pulse DBS are currently investigated. From computational modelling it has been proposed that short pulses might be more specific to activate selectively small-diameter, local axons around the electrode tip compared to more distant and thick myelinated axons of the capsula interna along the chronaxie of neuronal elements, resulting in decreased side effects and an increased therapeutic window^13^. Another recent computational modelling study proposes that short pulses reduce side effects by non-dose equivalent stimulation resulting in reduced spread of neural activation^29^. While there are incongruent computational results in terms of mechanisms of action of short pulses, the clinical effect of reducing side effects due to unintentional co-stimulation of neighbor structures has become an important strategy of DBS troubleshooting in the clinical routine. With respect to the parkinsonian tremor, we hypothesized that short pulse DBS would enhance the dentate-cerebellar-thalamic fiber selectivity and preserve the accidental co-stimulation of neighbor rubro-cerebellar fibers, optimizing the therapeutic window. In fact, we found efficient tremor reduction by oDBS30 with significant reduction of stimulation induced ataxia resulting in an enhanced therapeutic window.

Directional DBS was tested in intraoperative and postoperative settings in PD and ET patients^12,30–33^. The commercially available electrodes consist of leads with two middle rings divided into three segments. This electrode design enables the selection of only one segment resulting in axially defined asymmetric field restriction. Directional stimulation has therefore the potential to optimize spatially stimulation energy at the specific target fiber bundles, while preventing co-stimulation of fibers mediating unintentional side effects that may occur with omnidirectional stimulation. In previous studies using experimental lead designs during intraoperative recording^30,31^ and in postoperative studies implanting directional leads^32–34^, the therapeutic window could be enhanced by directional stimulation compared to omnidirectional stimulation. In particular, the stimulation amplitude for symptom relief was reduced, while the threshold for the induction of side effects was incremented. The real-world differences between directional and omnidirectional STN-DBS in PD patients were recently assessed in a large, prospective, international, multicenter, double-blind randomized crossover study^35^.

In the present study, the use of directional DBS was equally efficient in the reduction of the total tremor score compared to omnidirectional stimulation, still, dDBS60 was slightly inferior in the improvement of specific tremor subtypes as drawing tremor in PD patients. When comparing the use of directional DBS and short pulse DBS, dDBS seemed to be slightly inferior compared to short pulse DBS in the reduction of stimulation-induced side effects as ataxia, however one needs to consider the high amplitudes used in the study which might lower the spatial accuracy of dDBS60.

We found differential effects of the conventional and new stimulation modes between PD patients and ET patients in terms of tremor reduction and the extent of stimulation induced ataxia. The total tremor score seemed to be more improved in PD patients compared to ET patients by use of oDBS60 and oDBS30, though it must be considered that the tremor pattern was different between both patient groups. Besides, in PD patients the use of short pulses was even more effective in the reduction of stimulation-induced ataxia than in ET patients. These differential DBS effects might be explained by current pathophysiological assumptions of these different tremor entities in PD and ET patients. In PD, it is proposed that dysfunction within the basal ganglia-thalamo-cortical network could be the driver for the tremor onset, triggering then the cerebello-thalamic-cortical circuit which might be responsible for the tremor amplitude on its part^16^. In fMRI, PD tremor onset was correlated with activity changes in the putamen and globus pallidus, while PD tremor amplitude was correlating with cerebellum, thalamus and cortex^36^. In FDG-PETs of PD patients, the amount of tremor amplitude could be also correlated with activity changes in the dentate nucleus, rostral parts of the cerebellum and motor cortex^37^. In contrast, in ET patients the main pathology might be located within the cerebello-thalamo-cortical loop itself with a dysfunctional motor controller in the cerebellum^16^. In ET patients, cerebellar pathology has been described^38,39^. Thus, it was proposed that the direct generator of both tremor entities might be the cerebello-thalamo-cortical network, however the difference might be the way, this network is activated to oscillate.

Back to the findings of our study, one hypothesis could be that in PD patients the fiber bundles in this specific “bottleneck” within the cerebello-thalamo-cortical circuit are less affected by degenerative pathology than in ET^38,39^, since in PD the main pathology is located in the basal ganglia. Therefore, DBS might interfere with an oscillating, but otherwise functioning network in PD. In ET, the pathological degeneration of cerebellar outflow fibers might cutback and limit DBS therapeutical effects, particularly in terms of pre-existing ataxia due to cerebellar degeneration.

This study has certain limitations. The conclusions of our study are limited by the small sample size. However, the findings on short pulse stimulation are strong and fit to the existing literature. Though, the directional effects may be clearer in a larger study population. Furthermore, we used relatively high amplitudes between 2 and 4 mA, which can hamper the directionality of segmented stimulation^12^. Besides, we employed the TEED equation for the adjustment of amplitudes between the 30 µs and 60 µs DBS condition. This energy-equivalency might not be equal to a comparable spread of stimulation within the tissue^29^. The suprathreshold stimulation also limits the informative value on the tremor effect, as no effectiveness threshold was determined.

In summary, the application of new stimulation algorithms as short pulses or directional VIM/PSA-DBS might be beneficial in the tremor treatment of both PD and ET patients, particularly in the reduction of side effects as ataxia. In PD patients, the usage of the new stimulation forms seemed to be even more promising compared to ET, probably due to a different tremor phenomenology and pathophysiology.

## Supplementary Information

Supplementary Table 1.
