# Supplementary material for: Short pulse and directional thalamic deep brain stimulation have differential effects in parkinsonian and essential tremor
Source: Sci Rep. 2022 May 4;12:7251. doi: 10.1038/s41598-022-11291-9 (PMC9068767; doi:10.1038/s41598-022-11291-9)
Supplement: Supplementary file 1 — Supplementary Table 1. [file 41598_2022_11291_MOESM1_ESM.docx]

|  | **OFF** | | **oDBS60 ventral** | | **oDBS30 ventral** | | **oDBS60 dorsal** | | **oDBS30 dorsal** | | **dDBS60** | |
| --- | --- | --- | --- | --- | --- | --- | --- | --- | --- | --- | --- | --- |
|  | **PD** | **ET** | **PD** | **ET** | **PD** | **ET** | **PD** | **ET** | **PD** | **ET** | **PD** | **ET** |
| **FTMRS hand sub score** | 12.86±4.6 | 15.79±3.58 | 2.14±0.9 | 6.00±3.16 | 1.71±0.76 | 5.0±3.01 | 3.0±0.7 | 6.2±3.77 | 2±1.22 | 4.9±3.54 | 2.6±1.34 | 5.8±3.97 |
| **FTMRS**  **Rest tremor** | 3.57±0.79 | 0.07±0.27 | 0.14±0.38 | 0.0±0.0 | 0.14±0.38 | 0.0±0.0 | 0.0±0.0 | 0.0±0.0 | 0.0±0.0 | 0.0±0.0 | 0.0±0.0 | 0.0±0.0 |
| **FTMRS**  **Postural tremor** | 2.29±0.76 | 2.43±1.28 | 0.0±0.0 | 0.21±0.43 | 0.0±0.0 | 0.29±0.47 | 0.20±0.45 | 0.30±0.48 | 0.20±0.45 | 0.20±0.42 | 0.20±0.45 | 0.40±0.52 |
| **FTMRS**  **Action-/Intention tremor** | 0.43±0.53 | 3.07±1.00 | 0.0±0.0 | 0.21±0.43 | 0.0±0.0 | 0.29±0.47 | 0.0±0.0 | 0.40±0.70 | 0.0±0.0 | 0.0±0.0 | 0.0±0.0 | 0.10±0.32 |
| **FTMRS**  **Drawing** | 2.43±1.27 | 3.50±0.52 | 1.14±0.38 | 2.21±1.12 | 0.57±0.53 | 1.71±1.07 | 1.50±0.55 | 2.20±1.03 | 1.0±0.71 | 2.0±1.25 | 1.0±0.71 | 2.10±1.20 |
| **ICARS hand sub score** | 0.14±0.38 | 0.14±0.36 | 3.0±1.0 | 2.86±1.35 | 1.43±0.78 | 2,29±0.64 | 3.40±1.14 | 3.40±1.35 | 1.2±1.30 | 2.0±2.0 | 2.40±0.55 | 3.1±0.88 |

**Supplemental Table 1:** Overview of mean and standard deviation of tremor (FTMRS) score and ataxia score (ICARS hand subscore) in all stimulation conditions.
